# Predictors of Extubation Success in Patients with Middle Cerebral Artery Acute Ischemic Stroke

**DOI:** 10.4061/2011/248789

**Published:** 2011-10-01

**Authors:** Linda C. Wendell, Jonathan Raser, Scott Kasner, Soojin Park

**Affiliations:** ^1^Departments of Neurology and Neurosurgery, Rhode Island Hospital/Warren Alpert Medical School of Brown University, Providence, RI 02903, USA; ^2^Department of Neurology, Hospital of the University of Pennsylvania, Philadelphia, PA 19104, USA; ^3^Departments of Neurology, Neurosurgery, and Anesthesiology and Critical Care, Hospital of the University of Pennsylvania, Philadelphia, PA 19104, USA

## Abstract

*Introduction*. Stroke patients often meet respiratory guidelines for extubation, but uncertainty exists if patients will protect their airway due to impaired mental status. Patients with middle cerebral artery (MCA) acute ischemic stroke (AIS) might have specific predictors of successful extubation. *Methods*. Retrospective cohort of MCA AIS patients requiring intubation. *Results*. Thirty-seven MCA AIS patients were extubated successfully and ten failed extubation. Those who successfully extubated had higher extubation composite and eye response Glasgow Coma Scale (GCS) scores compared to those who failed (median 10T (IQR 9T–11T) versus 9.5T (8T–10T), *P* = 0.047, and 4 (3-4) versus 2.5 (1–3), *P* < 0.01). When adjusted for age, admission National Institutes of Health Stroke Scale score and laterality, patients with a GCS score ≥8T trended toward extubating successfully (OR 23.30 (CI 0.94–580.27), *P* = 0.055). *Conclusions*. The GCS score might be important in predicting successful extubation in MCA AIS patients. Further prospective study is warranted to better assess factors predictive of extubation outcome in stroke and other brain-injured patients.

## 1. Introduction

Acute ischemic stroke (AIS) can be complicated by cerebral edema and neurologic deterioration, requiring intubation and mechanical ventilation for respiratory support [1]. Once a stroke patient is mechanically ventilated, respiratory parameters guide the physician in determining when to discontinue mechanical ventilation and airway protection. Current guidelines from a collective task force including the American College of Chest Physicians, the American Association for Respiratory Care, and the American College of Critical Care Medicine recommend that extubation should be considered in patients with sufficient oxygenation, cardiovascular stability, improvement of the primary factor that led to respiratory failure and ventilatory dependence, and the patient's ability to initiate a breath [2]. Despite best practices, extubation failure occurs in 13–18% of critically ill patients [3–5]. 

Stroke and other brain-injured patients often meet respiratory guidelines for extubation, but the physician is uncertain if due to impaired mental status, a patient will be unable to protect his or her airway [6]. Weaning parameters (including vital capacity, minute ventilation, maximum inspiratory pressure, and the rapid shallow breathing index) can assist in determining a patient's ability to ventilate independently but do not predict a patient's ability to protect his or her airway. Additionally, respiratory weaning parameters within current guidelines are not predictive of extubation failure in neurocritical care patients intubated solely for neurologic reasons [7]. Given the limited data available to predict extubation success in critically ill neurologic patients, we hypothesized that patients with middle cerebral artery (MCA) AIS might have more specific predictors of successful extubation. 

## 2. Methods

A retrospective cohort of patients discharged between January 2004 and December 2008 with MCA AIS requiring intubation were studied. The institutional review board (IRB) of the Hospital of the University of Pennsylvania (HUP) approved the study prior to data collection. Patients were identified using the Clinical Effectiveness and Quality Improvement (CEQI) database. Patient characteristics were further identified using the Get With The Guidelines (GWTG) quality assessment database and review of patient charts. To be included, patients had to have acute ischemic infarct confined to the distribution of the MCA only and onset of stroke symptoms within 24 hours of admission or transfer. Patients with additional territories of stroke or primary intracerebral hemorrhage were excluded. Patients then were classified as having a failed extubation (defined as requiring reintubation within 48 hours) or a successful extubation. We excluded patients who had direct tracheostomy without extubation attempt, were extubated terminally, or died prior to an extubation attempt. 

Baseline clinical characteristics and past medical history were collected. Previous ischemic stroke was documented by history or by neuroimaging. Carotid stenosis was defined as ≥50% stenosis by history, carotid ultrasound, or vessel imaging using North American Symptomatic Carotid Endarterectomy Trial (NASCET) criteria. Atrial fibrillation or flutter was documented by history or by telemetry during hospitalization. Dyslipidemia was documented by history or by hospital laboratory testing demonstrating cholesterol ≥200 mg/dL, triglycerides >150 mg/dL, low-density lipoprotein (LDL) ≥160 mg/dL, or high-density lipoprotein (HDL) <40 mg/dL in men or <50 mg/dL in women [8]. Smoking history included current or former tobacco use. Admission National Institutes of Health Stroke Scale (NIHSS) scores, Glasgow Coma Scale (GCS) scores (at time of admission, intubation, and extubation), cortical stroke symptoms (including aphasia, neglect, and eyelid opening apraxia), and involvement of the basal ganglia on neuroimaging also were gathered for each patient. If NIHSS scores were not documented in the CEQI or GWTG databases or in the medical record, the scores were calculated using information from the chart. GCS scores were recorded from nursing flow sheets in the medical record. The intubated GCS score documented just prior to extubation with the patient off sedation was used for the extubation GCS score. We assigned a verbal score of 1T for intubated patients. Any analysis of an intubated GCS score was performed using the number only. As routine, nursing documented GCS scores as part of their neurologic exam every hour or every two hours. If a GCS score was not documented in the nursing flow sheet, the GCS score was calculated using information in the medical record. Day of intubation relative to stroke symptom onset was calculated for each patient. Day 0 was considered the first day of stroke symptom onset. While patients were intubated, sedation regimens varied to include both continuous infusions and intermittent administration of sedatives. Choice of sedation regimen was at the discretion of the treating physicians. The medical record was used to determine if patients developed pneumonia prior to or at time of extubation (defined by infiltrate on chest X-ray and decision by physician to treat with antibiotics). Total ventilator days and ventilator days prior to extubation attempt were recorded. Per respiratory protocol, patients were weaned to minimal pressure support ventilation and a 40% fraction of inspired oxygen (FiO_2_) prior to planned extubation. The decision to extubate was based on physician judgment. A minimum GCS score was not required for extubation. The GCS extubation score is the GCS score of sedation prior to extubation. Lengths of stay in the intensive care unit (ICU) and hospital and in-hospital mortality were also determined for all patients. 

Statistical analysis was completed using Stata SE 10 (College Station, Tex). Fisher's exact test was used to analyze categorical variables. The Wilcoxon sum rank test was used to analyze nonnormally distributed continuous variables. Univariate and multivariate logistic regressions were performed when appropriate. Significance was defined as *P* < 0.05.

## 3. Results

**Figure 1:**
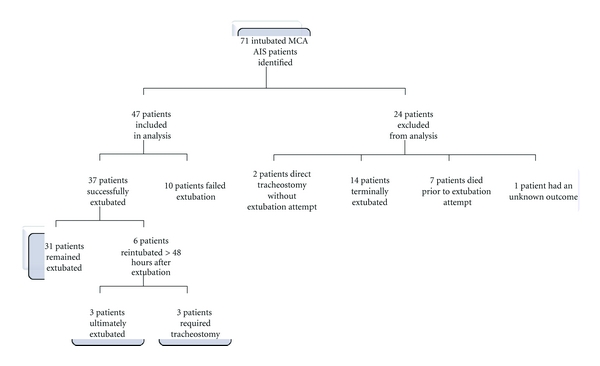
Extubation outcomes of intubated middle cerebral artery (MCA) acute ischemic stroke (AIS) patients identified.

Seventy-one patients with MCA AIS requiring intubation were identified (Figure 1). Thirty-seven patients (79%) were successfully extubated, and 10 patients (21%) failed extubation. These 47 patients were included for analysis. Four patients self-extubated, two patients were successfully extubated, and two patients failed extubation. Two patients had direct tracheostomy without extubation attempt, 14 patients were terminally extubated, and seven patients died prior to extubation attempt; these patients were excluded. Result of extubation is not known for one patient secondary to transfer to another facility within 48 hours of extubation. Six patients (16%) who initially had successful extubations required reintubation after 48 hours. Three (8%) of these patients ultimately received tracheostomies. 

Patients in the successful and failed extubation groups were similar with respect to age, gender, ethnicity, medical history, and baseline ambulatory status (Table 1). There were no differences between median admission GCS and NIHSS scores between those who successfully extubated and those who failed extubation (Table 2). Thirty-seven patients had left MCA strokes and 34 patients had right MCA strokes. Fifty-nine percent of patients who successfully extubated had infarct on the left compared to 30% of patients who failed extubation (*P* = 0.15). When adjusted for admission NIHSS score, acute left MCA AIS patients were more likely to extubate successfully (OR 8.57, CI 1.09–67.10, *P* = 0.04). The incidence of the cortical symptoms of aphasia, neglect, and eyelid opening apraxia was similar between those who had extubation success and those who failed extubation (Table 2). Both groups had a similar incidence of large strokes involving both superficial MCA territory and basal ganglia. There was no statistical difference in radiologic imaging between groups (Table 2).

**Figure 2:**
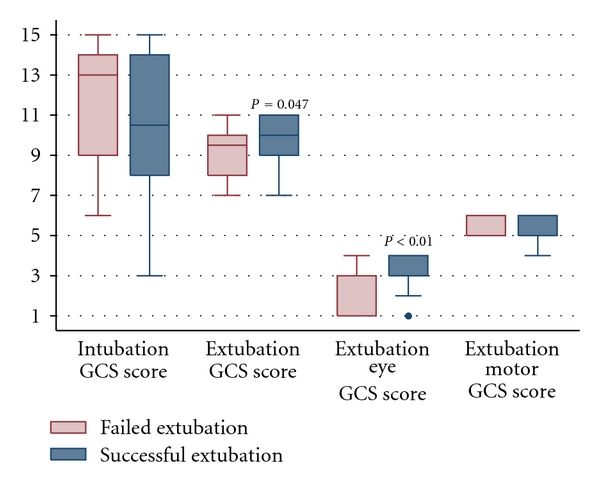
Glasgow Coma Scale (GCS) scores in middle cerebral artery acute ischemic stroke patients in whom extubation was successful versus failed.

Patients in whom extubation was successful versus failed had similar intubation GCS scores (median 10.5 (8–14) versus 13 (9–14), *P* = 0.81) (Figure 2). There was not a significant difference in incidence of intubation prior to admission or transfer to HUP or intubation for procedure between those who extubated successfully and those who failed (24% versus 10% and 38% versus 50%, resp.). Median day of intubation relative to stroke onset was day 1 (0–3) for those who successfully extubated and day 0 (0-1) for those who failed extubation (*P* = 0.21). Incidence of pneumonia was similar between groups (30% of those with a successful extubation versus 40% of those who failed extubation, *P* = 0.70). 

All patients had a cough and a rapid shallow breathing index <105 breaths/min/L at the time of the planned extubation attempt. Those patients who were successfully extubated had higher median extubation GCS scores than those who failed extubation (10T (9T–11T) versus 9.5T (8T–10T), *P* = 0.047) (Figure 2). When adjusted for age, admission NIHSS score, and laterality of stroke, patients with a GCS ≥8T trended toward extubating successfully (OR 23.30 (CI 0.94–580.27), *P* = 0.055). Patients with successful extubations also had higher median scores for best eye response of the GCS (4 (3-4) versus 3 (1–3), *P* < 0.01) (Figure 2). No difference in scores for best motor response of the GCS (median 6 (5-6) versus 6 (5-6), *P* = 0.95) was seen (Figure 2). The number of patients who were able to follow commands (motor GCS score of 6) was similar in the successful versus failed extubation groups (62% versus 60%, *P* = 1). 

**Figure 3:**
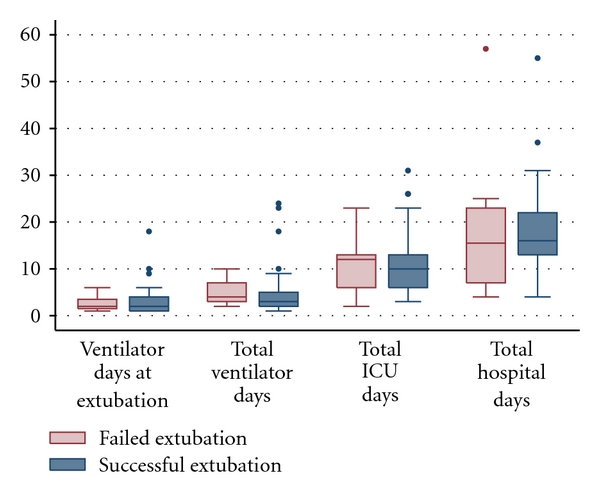
Outcomes of middle cerebral artery acute ischemic stroke patients in whom extubation was successful versus failed.

Those patients who were successfully extubated had similar total ventilator days (median 3 (2–5) versus 4 (3–7), *P* = 0.13). No differences were seen in median ventilator days at first extubation attempt (2 (1–4) versus 2 (1.5–3.5), *P* = 0.79), days in the ICU (10 (6–13) versus 12 (6–13), *P* = 0.91), or total hospital days (16 (13–22) versus 15.5 (7–23), *P* = 0.91) (Figure 3). A nonsignificant lower in-hospital mortality was seen in patients with extubation success (8% versus 20%, *P* = 0.29).

## 4. Discussion

Stroke and other brain-injured patients often meet respiratory guidelines for extubation, but the physician is uncertain if, due to impaired mental status, a patient will be unable to protect his or her airway [6]. Studies evaluating predictors of extubation success encompass a wide range of critically ill patients in medical and surgical ICUs [3–5]. Those studies that do focus on patients critically ill from neurologic illness also include patients with diverse diagnoses [6, 9]. Thus, predictors for extubation success are limited in brain-injured patients, including AIS patients. 

Our findings exhibit the use of the composite GCS score as a possible important predictor for successful extubation in MCA AIS patients. In this study, GCS scores ≥8T were associated with a trend toward extubation success in our population of MCA AIS patients. A higher GCS score previously has been shown to be a predictor for successful extubation in critically ill patients [5, 10], and daily screening for extubation readiness including a criterion for GCS ≥8 might be a better predictor for successful extubation in neurologic patients than physician judgment alone [9]. However, a recent study of patients critically ill from neurologic illness found no difference in GCS scores at time of extubation but a trend toward failed extubation in those with GCS scores between 7T and 9T [11]. While ischemic stroke patients were included in the latter study, the use of the GCS score as a predictor of extubation success specifically in MCA AIS patients has not been studied. Although ability to follow commands has been associated with extubation success in the neurologically ill patients [11], we found no difference in the ability to follow commands (as determined by a GCS motor score of 6) between those who successfully extubated and those who failed. Similarity between groups may be because all patients but one had high GCS motor scores of 5 (localization to stimulus) or 6 (following commands). In addition, the ability to follow commands may not be as important as the specific commands the patients is asked to follow [11]. The best eye response of the GCS also may be key in predicting extubation success in MCA AIS patients. It is not clear if the eye component of the GCS in isolation is more important than the composite GCS score or if both a high motor GCS score and a high eye GCS score are needed. 

Use solely of the GCS as a predictor for extubation success in MCA AIS patients can be problematic. No patients with a GCS score less than 7T were given a trial of extubation. Physician judgment regarding extubation readiness may be leading to a self-fulfilling prophecy that higher GCS scores are needed for extubation success. A previous study has demonstrated that patients with a GCS <5 can extubate successfully [6]. Another limitation of the GCS score is the limited and inconsistent scoring of the verbal response in intubated patients [12]. Given that the GCS was designed to assess for impaired consciousness, it does not account for specific neurologic impairment [13]. In MCA AIS patients, laterality of disease can result in eyelid opening apraxia (right-sided stroke symptom) or aphasia (left-sided stroke symptom), affecting composite GCS scores.

When adjusted for the admission NIHSS score, left MCA AIS patients were more likely to have extubation success than right MCA AIS patients. Those who failed extubation had higher nonsignificant incidence of neglect and eyelid opening apraxia. It may be that these right MCA-associated cortical signs influence extubation outcome and need further study. 

Several limitations to this study exist. The data collection was retrospective, and thus we were limited to information in the CEQI and GWTG databases and the medical record. Since many patients were unable to contribute to their own history taking when admitted with AIS, data regarding medical history may be underreported. All data points were not available for all patients. Reconstruction of the admission NIHSS, admission GCS, and intubation GCS scores from the chart was required for less than 25% of the patients. All patients but four had documented extubation GCS scores. Additionally, this study includes a small cohort of patients from a single institution. Specific protocols were not in place when assessing for extubation, and patients were extubated in multiple different units, which may have led to practitioner bias. A prospective study with an extubation protocol and a larger number of patients are needed to investigate further the utility of the GCS score in predicting extubation outcome.

## 5. Conclusions

Overall, there is a lack of data guiding extubation practices in AIS. Current national guidelines do not take neurologic dysfunction into consideration. Our study suggests that the GCS, particularly the eye response of the GCS, is predictive of extubation success. Further prospective study is warranted to better assess factors predictive of extubation outcome in stroke and other brain-injured patients.

## Figures and Tables

**Table 1:** Baseline characteristics of middle cerebral artery acute ischemic stroke patients in whom extubation was successful versus failed*.

	Successful (*n* = 37)	Failed (*n* = 10**)**
Age, median years (IQR)	62 (52–71)	51.5 (45–72)
Male	57% (21)	70% (7)
White	60% (21/35)	56% (5/9)
Previous stroke	41% (15)	50% (5)
Carotid stenosis	35% (13)	10% (1)
Coronary artery disease	27% (10)	40% (4)
Prosthetic heart valve	0% (2)	0% (0)
Atrial fibrillation or flutter	27% (10)	20% (2)
Congestive heart failure	24% (9)	40% (4)
Hypertension	71% (26)	60% (6)
Dyslipidemia	73% (27)	60% (6)
Peripheral vascular disease	5% (2)	0% (0)
Diabetes mellitus	30% (11)	20% (2)
Chronic obstructive pulmonary disease	16% (6)	10% (1)
Smoking history	59% (22)	78% (7/9)
Ambulating independently at baseline	97% (33/34)	90% (9)

*All *P* values are nonsignificant.

**Table 2:** Baseline clinical characteristics of middle cerebral artery acute ischemic stroke patients in whom extubation was successful versus failed*.

	Successful (*n* = 37)	Failed (*n* = 10)
Admission GCS^‡^ score, median (IQR)	11 (8–14)	11.5 (7–14)
Admission NIHSS^†^ score, median (IQR)	17 (12–22)	19 (14–21)
Left middle cerebral artery stroke	59% (22)	30% (3)
Aphasia	59% (22)	30% (3)
Neglect	38% (14)	60% (6)
Eyelid opening apraxia	5% (2)	10% (1)
Superficial and basal ganglia involvement	56% (20/36)	60% (6)
Superficial involvement only	36% (13/36)	40% (4)
Isolated basal ganglia infarct	8% (3/36)	0%

*All *P* values are nonsignificant.

^†^National Institutes of Health Stroke Scale.

^‡^Glasgow Coma Scale.
